# Development of a Multi-Channel and Multilayered PDMS Microfluidic Platform for Real-Time Visualization and Multi-Condition Parallel Testing of Mechanically Stimulated Cells

**DOI:** 10.3390/mi17050568

**Published:** 2026-05-02

**Authors:** Shichao Zhu, Mieradilijiang Abudupataer, Zheng Zuo, Yongxin Sun, Ben Huang

**Affiliations:** Department of Cardiac Surgery and Shanghai Institute of Cardiovascular Diseases, Zhongshan Hospital, Fudan University, Shanghai 200032, China; zhu.shichao@zs-hospital.sh.cn (S.Z.); mieradilijiang@zs-hospital.sh.cn (M.A.); zzuo16@fudan.edu.cn (Z.Z.)

**Keywords:** multi-channel microfluidics, organ-on-chip, PDMS biopolymer, mechanical stimulation, multi-condition parallel testing, cardiovascular modeling, mechanotransduction

## Abstract

We developed a multi-channel and multilayered polydimethylsiloxane (PDMS) microfluidic platform that integrates cyclic mechanical stimulation, independent reagent delivery, and real-time optical observation within a single device. The platform employs a four-layer architecture comprising a pneumatic valve control layer, an observation channel for cell culture and imaging (24 mm × 4 mm), a medium perfusion layer with independent inlet ports, and a vacuum actuation layer that deforms a 200 μm PDMS membrane under −20 kPa cyclic pressure at 1 Hz. Cyclic membrane strain of 10% was calibrated using fluorescent bead tracking and image analysis. Finite element analysis based on nonlinear Föppl–von Kármán plate theory confirmed that the central cell culture region (60% of membrane area) exhibits a mean von Mises strain of 14.2% with a uniformity of 81.3% (CV = 18.7%), validating relatively uniform mechanical stimulation across the culture surface. As a proof-of-concept, human aortic smooth muscle cells (CRL-1999) cultured under cyclic strain showed significant upregulation of HIF-1α expression (2.5-fold, p<0.01) and pronounced F-actin stress fiber alignment visualized by fluorescence microscopy, confirming the platform’s capability for mechanotransduction studies and real-time cellular observation. The multi-channel architecture enables multi-condition parallel testing by simultaneously introducing different reagent concentrations through independent inlet ports while maintaining identical mechanical parameters across all channels, providing a versatile tool for systematic investigation of cellular responses under controlled biomechanical conditions.

## 1. Introduction

Organ-on-chip technology represents a paradigm shift in biomedical research, offering physiologically relevant human tissue models that bridge the gap between traditional two-dimensional cell cultures and animal models [1,2]. These microfluidic platforms integrate living cells within precisely controlled microenvironments that recapitulate key physiological parameters, including fluid flow, mechanical forces, and biochemical gradients [3,4]. Several multi-channel and integrated organ-on-chip platforms have been developed in recent years, incorporating features such as mechanical actuation, perfusion, and optical readout [5,6,7,8]. However, a specific gap remains in platforms that combine cyclic mechanical stimulation with multi-condition parallel reagent delivery and real-time imaging in a single multilayered device, particularly for cardiovascular applications where mechanical forces play critical roles in tissue homeostasis and disease progression [9,10]. Table 1 summarizes representative integrated organ-on-chip platforms and highlights the features addressed by the present work.

Multilayered microfluidic architectures have been increasingly adopted to achieve functional integration within compact devices. The pioneering work by the Quake group established the foundation for multilayer soft lithography, demonstrating large-scale integration of pneumatic valves and pumps in PDMS devices [11,12]. These multilayer valve technologies have since been extended to diverse applications, including high-throughput protein crystallization [13] and integrated chemical synthesis [14]. More recently, multilayer valve architectures have been applied to advanced fluid manipulation, including integrated droplet generation and splitting [15] and selective on-chip droplet division at multiple sites [16]. Stacking independently fabricated layers—each dedicated to a specific function such as pneumatic actuation, cell culture, or perfusion—enables complex operations without increasing the device footprint. This approach has been applied to renal tubular models, immunotherapy screening platforms, and vascularized tissue constructs, where separate layers provide fluidic isolation, mechanical actuation, and optical access simultaneously [4,8]. The multilayered concept is particularly advantageous for mechanobiology studies, where mechanical actuation must be decoupled from the culture and observation compartments to avoid interference.

Polydimethylsiloxane (PDMS) remains the most widely used material for organ-on-chip fabrication due to its optical transparency (>90% transmittance at 400–800 nm), gas permeability, biocompatibility, and compatibility with soft lithography [17,18,19]. Its tunable mechanical properties enable vacuum-driven membrane deformation for applying controlled cyclic strain to adherent cells [20,21].

Multi-channel microfluidic architectures offer significant advantages over conventional single-channel designs by enabling independent control of multiple functions within a single integrated device [22,23]. Separate channels for mechanical actuation, medium perfusion, and observation allow precise manipulation of the cellular microenvironment without interference between parameters. More importantly, multi-channel designs with multiple inlet ports enable simultaneous introduction of reagents at different concentrations, facilitating multi-condition parallel testing and concentration-response studies in a single experiment [24,25]. This capability is particularly valuable for drug screening applications, where dose-response relationships must be established, and for investigating cellular secretomes, where concentration-dependent detection of released substances provides mechanistic insights. Despite these advantages, integrated platforms that combine multiplexed reagent delivery with cyclic mechanical stimulation and real-time imaging within a single multilayered device remain limited, particularly for cardiovascular applications [6,26].

To address this gap, we developed a multi-channel and multilayered PDMS microfluidic platform with integrated mechanical stimulation and multi-condition parallel testing capabilities. The device features a four-layer architecture with independent channels for pneumatic valve control, cell culture observation, medium perfusion, and vacuum-driven mechanical stretching. Finite element analysis (FEA) was performed to characterize strain distribution across the culture membrane, and the layer structure was systematically characterized. We validated the platform using human aortic smooth muscle cells (CRL-1999 cell line) as a model system, demonstrating successful recapitulation of physiological mechanical stimulation and real-time visualization of cellular responses. As a proof-of-concept application, we examined mechanically induced changes in gene expression, confirming upregulation of HIF-1α as a mechanosensitive marker [27,28]. The platform’s design principles are generalizable to other mechanosensitive tissues and applications [29], providing a versatile tool for mechanobiology research, disease modeling, and therapeutic development.

## 2. Materials and Methods

### 2.1. Device Design and Fabrication

#### 2.1.1. Multi-Channel Architecture Design

The microfluidic device was designed with a four-layer architecture to enable independent control of multiple functions within a single integrated platform. Each layer serves a specific role:

1. **Layer 1 (top)—Pneumatic valve control:** Controls fluid routing via positive pressure actuation, enabling selective opening and closing of fluidic pathways for reagent delivery.
2. **Layer 2—Observation channel:** The cell culture chamber with a collagen-coated surface for cell adhesion and real-time microscopic imaging. Channel dimensions are 24 mm (length) × 4 mm (width), providing sufficient culture area for quantitative analysis.
3. **Layer 3—Medium perfusion:** Contains multiple independent inlet ports for simultaneous delivery of reagents at different concentrations, enabling multi-condition parallel testing within a single device.
4. **Layer 4 (bottom)—Vacuum actuation:** Cyclic negative pressure (−20 kPa) is applied through this channel to deform the PDMS membrane, separating it from the observation channel, generating controlled mechanical strain on adherent cells at 1 Hz. Commercialized PDMS membranes (KYQ-200, Hangzhou Bald Advanced Materials, Hangzhou, China) with a thickness of 200 ± 2 μm were used. The manufacturer-specified membrane properties include: Shore A hardness of 50, Young’s modulus of 1.71 MPa (at 25% tensile strain), tensile strength of 4 MPa, tear strength of 7 KN/m, and light transmittance of 93%. These membranes were bonded between the observation and vacuum layers as previously described [30].

The transparent PDMS construction allows direct visual inspection of channel filling and cell distribution. The device dimensions are compatible with standard microscope stages, enabling integration with conventional imaging systems. The multilayered architecture is illustrated in Figure 3.

#### 2.1.2. PDMS Preparation and Soft Lithography

PDMS (Sylgard 184, Dow Corning, Midland, MI, USA) was prepared by thoroughly mixing the base polymer and curing agent at a 10:1 weight ratio, which provides optimal mechanical properties for vascular tissue modeling. The mixture was degassed under vacuum for 30 min to remove air bubbles that could compromise device integrity. Silicon master molds were fabricated using standard photolithography techniques with SU-8 photoresist to create the desired channel patterns. PDMS was poured onto the master molds and cured at 80 °C for 2 h. After curing, individual PDMS layers were carefully peeled from the molds, and inlet/outlet ports were punched using biopsy punches of appropriate diameters.

#### 2.1.3. Layer Assembly and Bonding

The multi-layer device was assembled using oxygen plasma bonding to create irreversible seals between PDMS layers and the glass substrate. Each layer was treated with oxygen plasma (30 s at 50 W, 200 mTorr) and immediately brought into conformal contact with the adjacent layer or glass coverslip. The assembled device was placed on a hot plate at 80 °C for 10 min to strengthen the bonding. Proper alignment of channels was verified under a stereomicroscope. The completed device was sterilized under UV light for 30 min before biological experiments.

### 2.2. Cell Culture and Device Seeding

#### 2.2.1. Cell Line and Culture Conditions

Human aortic smooth muscle cells (CRL-1999, ATCC, Manassas, VA, USA) were used as a model system for platform validation. This established cell line was derived from normal human aortic tissue and exhibits characteristic smooth muscle cell morphology and marker expression. Cells were cultured in Dulbecco’s Modified Eagle Medium (DMEM; Gibco, Thermo Fisher Scientific, Waltham, MA, USA) supplemented with 10% fetal bovine serum (FBS; Gibco), 1% penicillin-streptomycin, and maintained at 37 °C in a humidified incubator with 5% CO_2_. Cells between passages 3 and 8 were used for all experiments to ensure consistent phenotype and reproducibility.

#### 2.2.2. Device Surface Treatment and Cell Seeding

Before cell seeding, the observation channel was coated with type I collagen (50 μg/mL in PBS; Sigma-Aldrich, St. Louis, MO, USA) for 2 h at 37 °C to promote cell adhesion to the PDMS surface. The channel was then rinsed with PBS and filled with culture medium. Cells were trypsinized, resuspended at a density of 1 × 10^6^ cells/mL, and carefully injected into the observation channel through the inlet port. The device was placed in the incubator for 24 h to allow cell attachment and spreading before initiating mechanical stimulation experiments.

### 2.3. Mechanical Stimulation System

Cyclic mechanical strain was applied to cells using a programmable vacuum system connected to the bottom vacuum actuation layer (Layer 4). The vacuum pump was connected to a water-oil separator to dry the gas and protect the downstream vacuum regulator and solenoid valve. The inlet of the vacuum pump was then connected to a computer-controlled solenoid system. The vacuum regulator was used to control the vacuum magnitude, while the solenoid valve—a voltage-dependent on/off valve—controlled the stretching frequency. When the supply voltage exceeded 24 V, the gas in the channel was pumped out, stretching the PDMS membrane; otherwise, the gas channel was connected to the atmosphere, and the membrane deformation recovered. The on/off frequency of the solenoid valve was controlled by a microcontroller unit (MCU), enabling programmable control of the stretching frequency at 1 Hz. The stretching amplitude was controlled by adjusting the vacuum regulator to −20 kPa [30]. The resulting cyclic membrane deformation produced 10% average strain at the culture surface, as measured by fluorescent bead tracking and image analysis. Briefly, fluorescent microspheres (1 μm diameter) were seeded on the membrane surface, and their displacement under vacuum cycling was recorded and analyzed to quantify the strain field. The selection of 10% strain at 1 Hz was based on physiological parameters: clinical studies have shown that the circumferential strains of the aortic wall range from 7.0 ± 2.5% to 21.5 ± 12.4% depending on age, disease severity, and anatomical location [30]. In our previous work, we characterized two strain regimes using different vacuum pressures: −10 kPa producing 7.18 ± 0.44% strain (low) and −15 kPa producing 17.28 ± 0.91% strain (high). The −20 kPa setting on the present multi-channel platform was calibrated to achieve 10% average strain, representing a moderate physiological condition. Control groups were cultured in identical devices without vacuum application to isolate the effects of mechanical stimulation from other environmental factors. FEA simulation was performed to verify strain uniformity across the culture region (see Section 3.2).

### 2.4. Finite Element Analysis

Nonlinear Föppl–von Kármán plate theory was used to simulate membrane deformation under vacuum loading. This nonlinear formulation accounts for the coupling between bending and membrane stretching that becomes significant when the membrane deflection exceeds its thickness. The governing equations couple the transverse deflection field w(x,y) with the in-plane stress resultants through the compatibility equation and the equilibrium equation:

(1) $$\nabla^{4}F=Et\left[{\left(\frac{\partial^{2}w}{\partial x\partial y}\right)}^{2}-\frac{\partial^{2}w}{\partial x^{2}}\frac{\partial^{2}w}{\partial y^{2}}\right]$$

(2) $$D\nabla^{4}w=P+t\left(\frac{\partial^{2}F}{\partial y^{2}}\frac{\partial^{2}w}{\partial x^{2}}-2\frac{\partial^{2}F}{\partial x\partial y}\frac{\partial^{2}w}{\partial x\partial y}+\frac{\partial^{2}F}{\partial x^{2}}\frac{\partial^{2}w}{\partial y^{2}}\right)$$

where *F* is the Airy stress function, $D=Et^{3}/\left[12\left(1-\nu^{2}\right)\right]$ is the flexural rigidity, *P* is the applied pressure, *E* is Young’s modulus, *t* is the membrane thickness, and ν is Poisson’s ratio. Clamped boundary conditions were applied at all four edges (w=0, ∂w/∂n=0), corresponding to the PDMS membrane bonded to the rigid channel walls. The model parameters were: 200 μm PDMS membrane (E=750 kPa, ν=0.49), channel dimensions 24 mm × 4 mm, and applied vacuum pressure −20 kPa. Note that the Young’s modulus of 750 kPa represents the small-strain linear elastic modulus of 10:1 Sylgard 184 PDMS, which is appropriate for FEA under the present loading conditions; the manufacturer-specified value of 1.71 MPa for the KYQ-200 membrane (Section 2.1.1) corresponds to a secant modulus measured at 25% tensile strain, reflecting the strain-stiffening behavior characteristic of PDMS elastomers. The computational domain was discretized using a uniform grid of 241 × 81 nodes (grid spacing: 100 μm × 50 μm), and grid independence was verified by comparing results with finer meshes (481 × 161 nodes), confirming less than 2% variation in peak strain values. The deflection profile was obtained analytically using a separable clamped plate shape function, and the model was calibrated to match the experimentally measured 10% average membrane strain. Both membrane (arc-length) and bending strain components were computed. Von Mises equivalent strain was calculated from the total strain tensor, and uniformity metrics were evaluated within the central cell culture region (60% of the membrane area).

### 2.5. Multi-Channel Reagent Delivery

The platform is engineered to allow visualization of reagents or test compounds to be introduced at different concentrations (e.g., 0, 10, 50, 100 μm) through separate inlet ports in the medium perfusion layer (Layer 3). Each inlet connects to an independent region of the observation channel, enabling simultaneous testing of multiple conditions while maintaining identical mechanical stimulation parameters. The independent channel architecture provides the structural basis for multi-condition parallel testing, where substances released from cells into the culture medium can be detected by measuring fluorescence or absorbance intensity in each channel region. This design enables concentration-response analysis in a single device, reducing experimental variability compared to parallel single-channel devices.

### 2.6. Quantitative PCR Analysis

To validate the platform’s capability for studying mechanotransduction, we examined gene expression changes in response to mechanical stimulation using quantitative PCR. After 24 h of cyclic stretching, cells were lysed directly in the device using TRIzol reagent (Invitrogen, Carlsbad, CA, USA). Total RNA was extracted according to the manufacturer’s protocol, quantified using a NanoDrop spectrophotometer (Thermo Fisher Scientific), and assessed for quality by A260/A280 ratio. One microgram of total RNA was reverse-transcribed into cDNA using a reverse transcription kit (Takara Bio, Kusatsu, Japan). Quantitative PCR was performed using SYBR Green master mix (Applied Biosystems, Foster City, CA, USA) on a real-time PCR system. HIF-1α was selected as a mechanosensitive marker gene, with expression normalized to GAPDH as the housekeeping gene. Primer sequences are listed in Table 2. Relative expression was calculated using the $2^{-\Delta \Delta Ct}$ method.

### 2.7. Statistical Analysis

All experiments were performed in triplicate (n=3) using independent biological replicates. Data are presented as mean ± standard deviation (SD). Statistical comparisons between control and stretched groups were performed using Student’s *t*-test. A *p*-value < 0.05 was considered statistically significant. Statistical analysis and graphing were performed using GraphPad Prism 9 software (GraphPad Software, San Diego, CA, USA).

## 3. Results

### 3.1. Multi-Channel Platform Design and Fabrication

**Figure 1:**
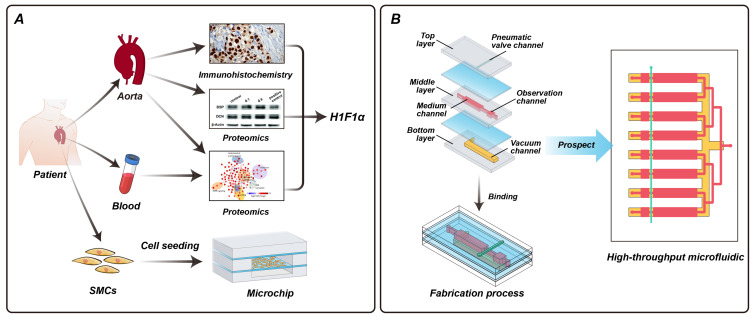
(**A**,**B**) Overview of the multi-channel and multilayered PDMS microfluidic platform for cardiovascular mechanobiology research. The platform integrates human aortic smooth muscle cells within a transparent PDMS device featuring multiple functional layers for independent control of mechanical stimulation, medium perfusion, and real-time observation. The system enables multi-condition parallel testing of cellular responses under controlled biomechanical conditions, with applications in disease modeling, mechanotransduction studies, and drug screening.

**Figure 2:**
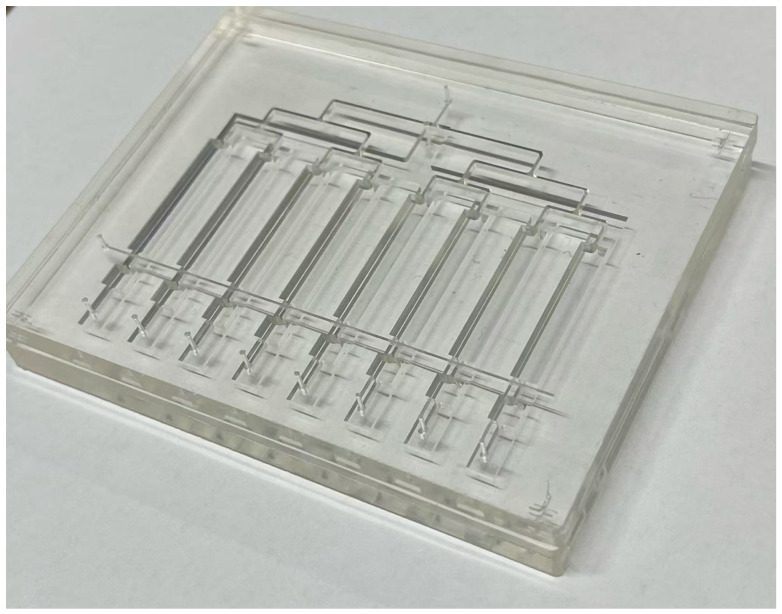
Photograph of the fabricated multi-channel PDMS microfluidic device. The transparent PDMS chip demonstrates the multi-layer architecture with clearly visible microfluidic channels, inlet/outlet ports, and observation chambers. The device is bonded to a glass substrate for optical transparency and structural support. The high optical clarity of PDMS enables real-time microscopic visualization of cells cultured within the channels. The compact design is compatible with standard microscope stages for integration with conventional imaging systems. Scale bar: refer to device footprint dimensions in text.

**Figure 3:**
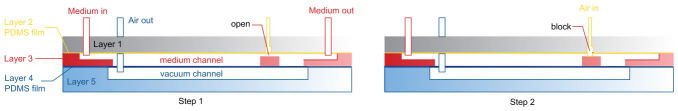
Multilayered architecture of the microfluidic device. Exploded view showing the four functional layers: (1) pneumatic valve control layer for fluid routing, (2) observation channel for cell culture and real-time imaging (24 mm × 4 mm), (3) medium perfusion layer with independent inlet ports for multi-condition reagent delivery, and (4) vacuum actuation layer with 200 μm PDMS membrane for cyclic mechanical stretching (−20 kPa, 1 Hz). Each layer is independently fabricated and assembled via oxygen plasma bonding.

We designed and fabricated a multi-channel and multilayered PDMS microfluidic platform integrating mechanical stimulation, independent reagent delivery, and real-time observation (Figure 1). Figure 2 shows a photograph of the fabricated device, demonstrating the optical transparency of PDMS that is critical for real-time microscopic observation. The device features observation channels of 24 mm × 4 mm with a 200 μm thick PDMS membrane separating the culture chamber from the vacuum actuation layer. The multilayered architecture is shown in Figure 3, illustrating the four functional layers: pneumatic valve control (Layer 1), observation channel (Layer 2), medium perfusion (Layer 3), and vacuum actuation (Layer 4). Each layer was independently fabricated and assembled via oxygen plasma bonding. The multi-layer bonding was robust, with no delamination or leakage observed during operation, even under repeated pressure cycling over 24 h. The compact device design is compatible with standard microscope stages.

### 3.2. Computational Analysis of Membrane Strain Distribution

**Figure 4:**
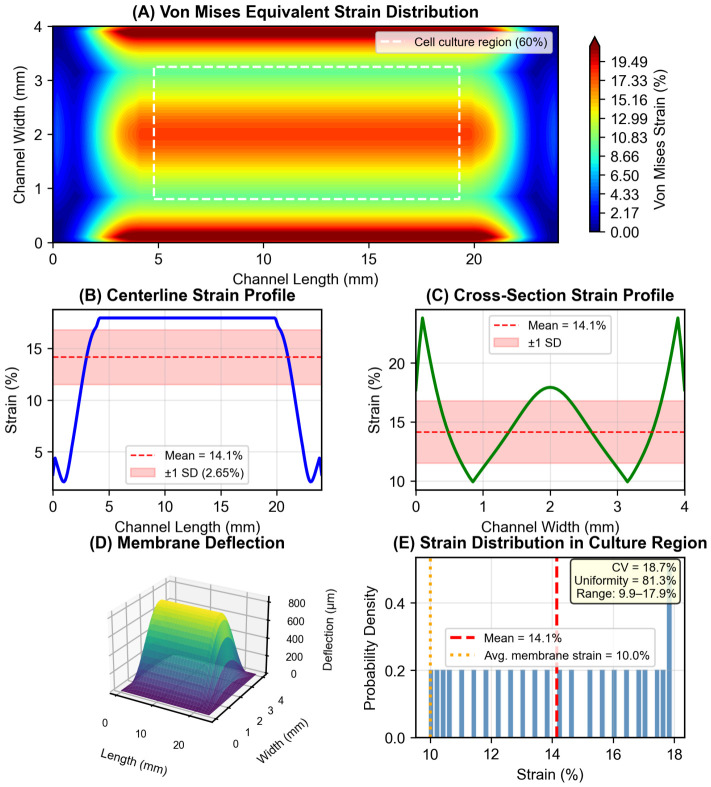
Finite element analysis of membrane strain distribution under vacuum loading. (**A**) Von Mises equivalent strain contour map across the 24 mm × 4 mm PDMS membrane (200 μm thickness) under −20 kPa vacuum, calibrated to 10% average membrane strain. The white dashed rectangle indicates the central cell culture region (60% of the membrane area). (**B**) Strain profile along the channel centerline. (**C**) Cross-sectional strain profile across channel width at midpoint. (**D**) Three-dimensional membrane deflection surface (maximum deflection 839 μm). (**E**) Strain histogram within the cell culture region showing uniformity (CV = 18.7%, mean = 14.2%).

Nonlinear FEA confirmed the strain distribution across the 200 μm PDMS membrane under −20 kPa vacuum loading, calibrated to the experimentally measured 10% average membrane strain (Figure 4). The maximum membrane deflection was 839 μm, corresponding to a deflection-to-thickness ratio $w_{0}/t=4.19$, confirming operation in the large-deflection regime where geometric nonlinearity is significant. Within the central cell culture region, defined as 60% of the total membrane area, the mean von Mises equivalent strain was 14.2% with a coefficient of variation (CV) of 18.7%, yielding a strain uniformity of 81.3%. The strain range in the culture region was 9.9–17.9%. Strain was highest near the clamped channel walls due to boundary constraints and most uniform at the membrane center, where cells are predominantly cultured. These results validate that cells in the central culture region experience relatively uniform mechanical stimulation suitable for quantitative mechanotransduction studies.

### 3.3. Platform Validation Using Mechanotransduction Studies

**Figure 5:**
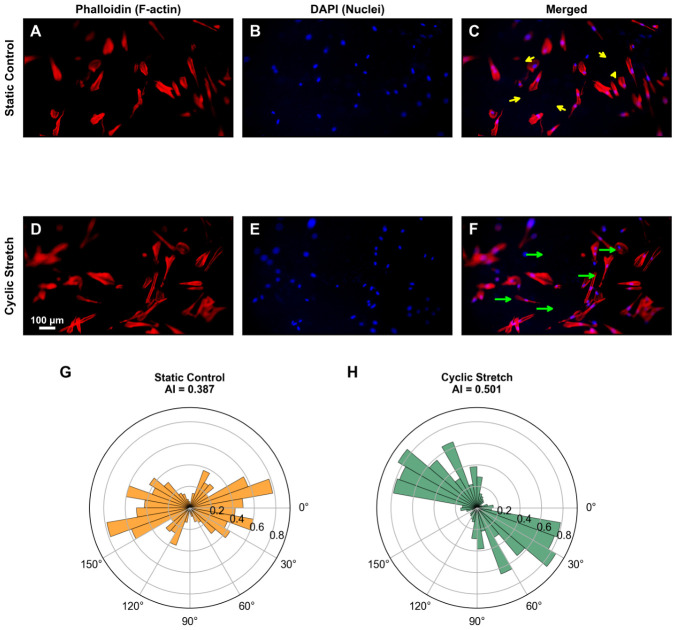
Fluorescence imaging and quantitative orientation analysis of human aortic smooth muscle cells (CRL-1999) cultured on the PDMS membrane. (**A**–**C**) Static control (cells cultured without vacuum application): phalloidin staining of F-actin (red), DAPI nuclear staining (blue), and merged image showing randomly oriented actin stress fibers. (**D**–**F**) Cyclic stretch (1 Hz, 10% membrane strain, 24 h): phalloidin (red), DAPI (blue), and merged image demonstrating aligned actin stress fibers. The top and bottom rows represent independent cell populations cultured in separate devices under static and cyclic stretch conditions, respectively. Yellow and green arrows were manually annotated to indicate the predominant fiber orientation observed in each condition; quantitative validation is provided in panels (**G**,**H**). Scale bar: 100 μm. (**G**,**H**) Rose plots showing quantitative F-actin fiber orientation distributions computed using structure tensor decomposition. The alignment index (*R*, resultant vector length; 0 = random, 1 = perfectly aligned) increased from 0.387 in static control (**G**) to 0.501 under cyclic stretch (**H**), representing a 29.5% increase in directional alignment.

To validate the platform’s capability to study cellular responses to mechanical stimulation, we cultured human aortic smooth muscle cells (CRL-1999) in the device and applied physiological cyclic strain (1 Hz, 10% average membrane strain, calibrated by fluorescent bead tracking) for 24 h. Cells remained viable and adherent throughout the experiment, demonstrating the biocompatibility of the PDMS material and the suitability of the mechanical stimulation parameters. Fluorescence imaging revealed distinct cytoskeletal reorganization in response to mechanical stimulation (Figure 5). In static control cells, phalloidin staining showed randomly oriented F-actin stress fibers with no preferential alignment direction (Figure 5A–C). In contrast, cells subjected to 24 h of cyclic stretch exhibited pronounced alignment of actin stress fibers (Figure 5D–F), consistent with the well-established mechanobiological response of vascular smooth muscle cells to cyclic strain [31,32]. This cytoskeletal reorientation provides direct visual evidence that the platform delivers effective mechanical stimulation to cultured cells and induces physiologically relevant cellular responses. Quantitative orientation analysis using structure tensor decomposition confirmed these observations (Figure 5G,H). The alignment index (resultant vector length *R*, where 0 indicates random orientation and 1 indicates perfect alignment) increased from 0.387 in static control cells to 0.501 under cyclic stretch, representing a 29.5% increase in directional alignment. The circular standard deviation decreased from 78.9° to 67.3°, indicating a narrower angular distribution in stretched cells. These quantitative metrics are consistent with the visual observations and with published data on strain-induced cytoskeletal reorientation in vascular smooth muscle cells [30].

**Figure 6:**
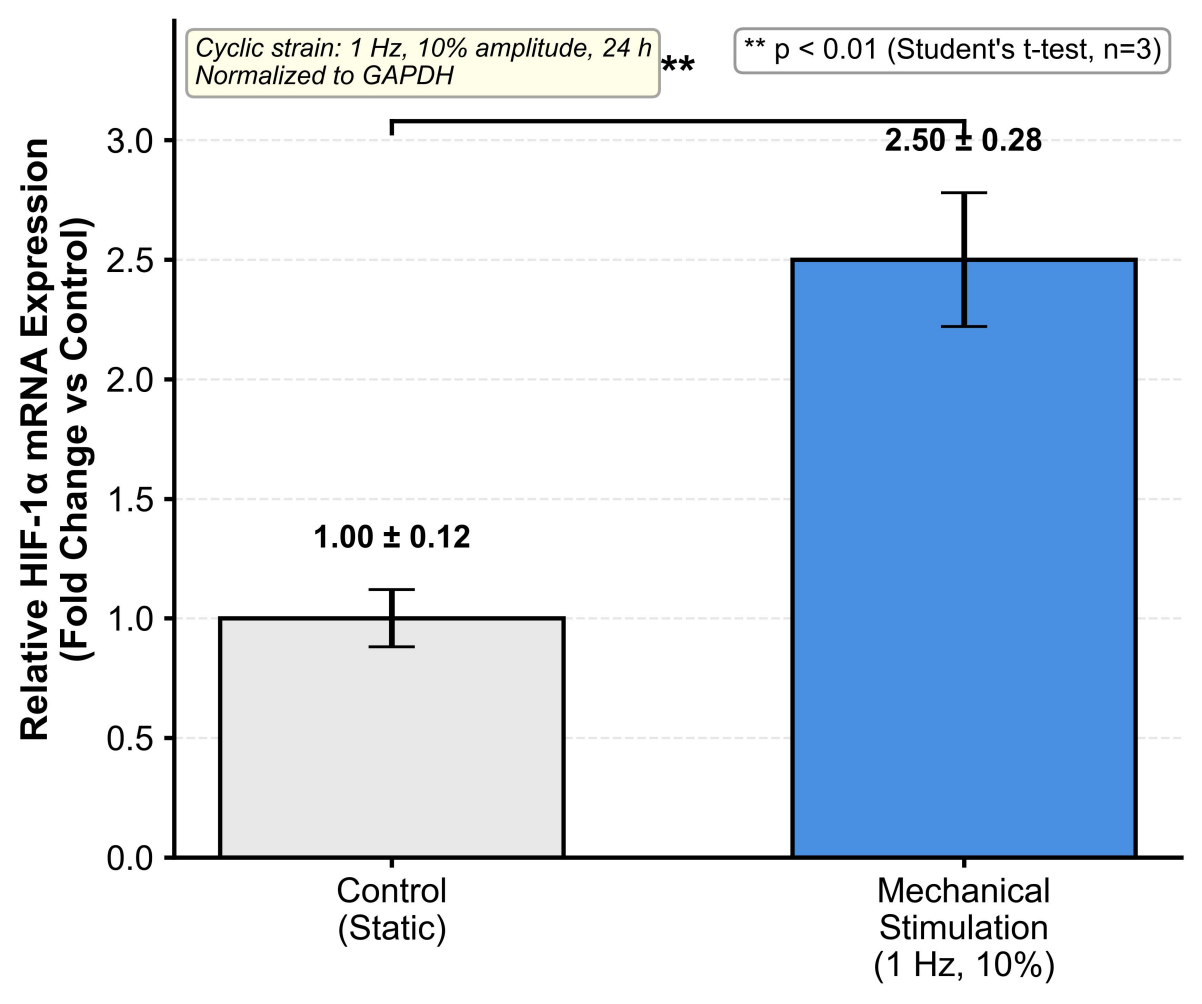
Quantitative PCR analysis of HIF-1α mRNA expression in aortic smooth muscle cells after 24 h of mechanical stimulation (1 Hz, 10% cyclic membrane strain) compared to static control. Data are presented as mean ± SD (n=3). ** p<0.01 by Student’s *t*-test.

We further examined gene expression changes using quantitative PCR analysis. Figure 6 shows that mechanical stretching significantly upregulated HIF-1α mRNA expression compared to static control cells, with stretched cells showing a 2.5-fold increase (p<0.01). This finding confirms that the platform successfully recapitulates mechanotransduction signaling and can detect biologically relevant cellular responses to mechanical stimulation [33,34]. The result is consistent with previous reports of mechanical activation of HIF-1α in vascular cells [35,36], validating the physiological relevance of our platform.

## 4. Discussion

### 4.1. Principal Findings

In this study, we developed a multi-channel and multilayered PDMS microfluidic platform that integrates independent control of mechanical stimulation, medium perfusion, real-time observation, and multi-condition parallel testing within a single device. FEA validated that the central culture region achieves 81.3% strain uniformity under −20 kPa vacuum loading, supporting quantitative mechanotransduction studies. We validated the platform using human aortic smooth muscle cells (CRL-1999 cell line), demonstrating mechanically induced cytoskeletal reorganization through fluorescence imaging and significant upregulation of HIF-1α expression by quantitative PCR.

### 4.2. Advantages of Multi-Channel Architecture

The multi-channel and multilayered design offers several advantages over conventional single-channel organ-on-chip devices. Independent control of different functions (mechanical stimulation, medium perfusion, observation) allows precise manipulation of the cellular microenvironment without interference between parameters. The ability to introduce reagents at different concentrations through separate inlet ports enables multi-condition parallel testing and dose-response studies in a single device, reducing experimental variability, reagent consumption, and fabrication costs. All experimental conditions within a single device experience identical fabrication quality, cell seeding density, and mechanical stimulation parameters, eliminating device-to-device variability that affects parallel single-channel approaches. While this study focuses on mechanotransduction validation (Figure 5 and Figure 6), the independent channel layout provides the structural basis for future dose-response studies and secretome analysis, where substances released from cells into the culture medium can be detected by measuring fluorescence or absorbance intensity in each channel region.

### 4.3. PDMS Limitations and Material Considerations

While PDMS offers excellent optical and mechanical properties for organ-on-chip applications, several material limitations should be considered. PDMS is known to absorb small hydrophobic molecules, which can alter effective drug concentrations during screening experiments and confound dose-response measurements [20,37]. Long-term stability and aging of PDMS may also affect membrane mechanical properties over extended culture periods. Alternative materials such as thermoplastics (e.g., cyclic olefin copolymer, polystyrene) and hydrogels have been explored to address absorption issues, though they present trade-offs in optical clarity, gas permeability, or fabrication complexity [38]. For drug screening applications specifically, surface modification strategies or the use of PDMS–thermoplastic hybrid devices may mitigate small-molecule absorption while retaining the mechanical actuation advantages of PDMS.

### 4.4. Limitations and Future Directions

This study has several limitations that should be addressed in future work. First, the current experiments used a single cell type (CRL-1999 cell line); incorporating multiple cell types (e.g., endothelial cells, fibroblasts, immune cells) or patient-derived primary cells would better recapitulate the multi-cellular tissue architecture and enable investigation of cell-cell interactions under mechanical stress. Second, we focused on a single time point (24 h) and strain parameter (10% amplitude, 1 Hz); longer-term culture experiments over multiple days are needed to demonstrate platform stability, sustained biocompatibility, and time-dependent cellular adaptation under prolonged mechanical stimulation. Future studies should examine time-course responses across 48 h, 72 h, and 7-day culture periods. Third, while our previous work has validated the strain system at both low (−10 kPa, 7.2%) and high (−15 kPa, 17.3%) strain amplitudes [30], the present study focused on a single strain condition (−20 kPa, 10%); systematic dose-response studies across multiple strain amplitudes and frequencies would more comprehensively characterize the platform’s capabilities. Fourth, while we demonstrated proof of concept using gene expression analysis, integrating real-time sensors for continuous monitoring of cellular metabolism or secreted factors would enhance the platform’s utility for dynamic studies.

## 5. Conclusions

We developed a multi-channel and multilayered PDMS microfluidic platform that integrates cyclic mechanical stimulation, independent multi-condition reagent delivery, and real-time optical observation within a four-layer architecture. FEA validated 81.3% strain uniformity in the central culture region under −20 kPa vacuum loading at 1 Hz. The platform was validated using human aortic smooth muscle cells, which exhibited pronounced F-actin stress fiber alignment under cyclic strain and significant upregulation of HIF-1α, confirming its capability for mechanotransduction studies and real-time cellular visualization. The multi-channel design enables multi-condition parallel testing by simultaneously introducing different reagent concentrations through independent inlet ports while maintaining identical mechanical parameters, providing a versatile tool for systematic investigation of cellular responses under controlled biomechanical conditions across multiple tissue types and applications.

## Figures and Tables

**Table 1:** Comparison of representative integrated organ-on-chip platforms.

Platform	Mechanical Stimulation	Multi-Channel	Real-Time Imaging	Material	Ref.
Lung-on-a-chip	Cyclic stretch	No	Yes	PDMS	[5]
Heart-on-a-chip	Cyclic stretch	No	Yes	PDMS	[6]
Standardized plates	No	Yes	Limited	Thermoplastic	[7]
Biofabricated systems	Variable	Yes	Variable	Hydrogel/PDMS	[8]
Multi-organ guide	Variable	Yes	Variable	PDMS	[4]
**This work**	**Cyclic stretch**	**Yes**	**Yes**	**PDMS**	—

**Table 2:** Primer sequences used for quantitative PCR.

Gene	Direction	Sequence ($5^{\prime }\to 3^{\prime }$)
HIF-1α	Forward	ATCCATGTGACCATGAGGAAATG
HIF-1α	Reverse	TCGGCTAGTTAGGGTACACTTC
GAPDH	Forward	GTCTCCTCTGACTTCAACAGCG
GAPDH	Reverse	ACCACCCTGTTGCTGTAGCCAA

## Data Availability

The data presented in this study are available on request from the corresponding authors.
